# Exercise Training Across the Hypertensive Heart Disease Continuum: Clinical Evidence and Implications for Prescription

**DOI:** 10.3390/jcdd13090441

**Published:** 2026-09-07

**Authors:** Xiongkai Yu, Chi Zhang, Lu Liu, Huimin Chen

**Affiliations:** 1 Department of Cardiology, The Second Affiliated Hospital, School of Medicine, Zhejiang University, Hangzhou 310009, China; 2 State Key Laboratory of Transvascular Implantation Devices, Hangzhou 310009, China; 3 Heart Regeneration and Repair Key Laboratory of Zhejiang Province, Hangzhou 310009, China

**Keywords:** hypertension, hypertensive heart disease, exercise training, exercise prescription, left ventricular hypertrophy, diastolic dysfunction, heart failure with preserved ejection fraction, arterial stiffness, autonomic nervous system

## Abstract

Arterial hypertension is the leading driver of hypertensive heart disease (HHD), the cardiac structural or functional injury attributable to sustained pressure overload, which promotes left ventricular hypertrophy and provides the substrate for incident heart failure, especially heart failure with preserved ejection fraction (HFpEF). Antihypertensive pharmacotherapy lowers blood pressure (BP) and regresses hypertrophy, yet residual cardiovascular risk persists, and exercise training may add to what drug therapy achieves. HHD progresses through interwoven autonomic, vascular, structural, and diastolic mechanisms, and the benefits of training are uneven across them: functional capacity, BP, endothelial function, and autonomic indices improve within typical trial durations, whereas resting diastolic indices, left ventricular mass, and myocardial fibrosis markers change inconsistently. Carotid–femoral pulse wave velocity is itself sensitive to distending pressure, so a fall does not by itself establish structural arterial remodeling. Small hypertensive trials support favorable geometry, and animal models support antifibrotic mechanisms, but in predominantly hypertensive HFpEF cohorts, capacity gains often occur without durable resting diastolic reversal or mortality benefit. By pairing each hypertension-specific mechanism with the corresponding human evidence, the present narrative review concludes that exercise training is a beneficial adjunct to antihypertensive therapy and to broader risk-factor management, while reverse remodeling and clinical-event reduction remain incompletely proven. Phenotype-guided trials with sensitive imaging, exercise-load endpoints, and hard outcomes are needed.

## 1. Introduction

Arterial hypertension affects on the order of a billion adults worldwide and ranks among the leading causes of cardiovascular death [1]. The heart bears a disproportionate share: sustained pressure overload remodels the myocardium into hypertensive heart disease (HHD), the substrate that links hypertension to most incident heart failure. In the Framingham cohort, hypertension preceded heart failure in roughly nine of every ten patients [1,2]. HHD denotes the cardiac structural and functional alterations attributable to sustained hypertension, spanning a clinical range from concentric remodeling and left ventricular hypertrophy (LVH), through diastolic dysfunction, to symptomatic heart failure. The commonest syndrome of HHD, heart failure with preserved ejection fraction (HFpEF), is also the least responsive to pharmacological treatment [2]. Once regarded as a compensatory response, pressure-overload hypertrophy is now recognized as maladaptive: it stiffens the ventricle, degrades performance, predisposes to arrhythmia, and is itself prognostically adverse [3]. Antihypertensive pharmacotherapy lowers blood pressure (BP) and regresses LVH, and normalizing an abnormal geometry improves survival [4]. The magnitude of the blood-pressure reduction nonetheless does not correlate with the magnitude of LVH regression [5], which leaves room for treatments that reach the same disease process by additional routes [6].

Over the past decades, structured exercise training, delivered alone or within cardiac rehabilitation, has emerged as a candidate intervention for the hypertensive trajectory. Long approached with caution in patients with LVH and an exaggerated blood-pressure response [7], training has become a mechanism-targeted adjunct [8] whose case rests less on blood-pressure lowering alone than on the breadth of mechanisms recruited. Training lowers office and ambulatory blood pressure across hypertensive populations, with reductions in resistant hypertension that rival device-based therapy [9,10]. Modality matters less than once assumed: high-intensity interval training (HIIT) and moderate continuous training (MCT) raise aerobic capacity and lower pressure comparably in cardiometabolic populations, while isometric work ranks highest for blood-pressure reduction in network meta-analysis [11,12]. Observational data extend the case across the disease course. Each increment of one metabolic equivalent (MET) in cardiorespiratory fitness is associated with roughly 10% lower incident hypertension in initially normotensive adults [13], and with about 22% lower all-cause mortality once hypertension is established [14]. The clinical question has shifted from whether exercise is safe in HHD to how it should be dosed and which phenotype benefits most.

The present review asks where, along the HHD continuum, exercise training modifies the disease process and where it improves a surrogate signal only. Four levels are examined: autonomic and neurohormonal drive, endothelial function and arterial stiffness, left ventricular remodeling, and diastolic function with hypertensive HFpEF. They are presented in that order for exposition. The mechanisms in fact form a feed-forward circuit. The neurohormonal mediators act on the myocardium directly as well as through afterload; arterial stiffening and ventricular remodeling advance in parallel. Stiffened arteries deform the baroreceptors and re-excite the sympathetic outflow that began the cycle [15,16]. Current society statements address exercise in hypertension and in HFpEF by modality and by syndrome [8,17]. Recent narrative reviews cover exercise in HHD and diastolic dysfunction by mechanism [6,18] without separating conclusions by the phenotype of the cohort that supports them. The present review is organized instead by mechanism and by the strength of the evidence available at each level. A conclusion resting on hypertension-specific trials is therefore not presented as equivalent to one resting on predominantly hypertensive HFpEF cohorts or on preclinical models. Evidence anchored to a hypertensive etiology is accordingly kept separate from evidence merely compatible with it. Non-hypertensive entities such as obesity- or age-related HFpEF, infiltrative and hypertrophic cardiomyopathies, and valvular disease enter only as differentials [5,18].

## 2. Scope and Methods

This critical narrative review was developed from PubMed searches combined with backward and forward citation tracing. Core and endpoint-specific searches were last rerun on 10 July 2026, and representative saved search strings are reported in Supplementary Materials Table S2. Search concepts combined arterial hypertension, HHD, left ventricular (LV) hypertrophy or remodeling, and diastolic dysfunction or HFpEF with exercise training, physical activity, exercise prescription, or cardiac rehabilitation. Endpoint searches addressed autonomic regulation, vascular function and stiffness, cardiac structure and fibrosis, diastolic reserve, functional capacity, clinical events, dose, safety, and delivery. Society statements, systematic reviews and meta-analyses, randomized and controlled training studies, and large observational cohorts were used according to the question each could answer. Preclinical studies were retained only for pressure-overload mechanisms that cannot be tested directly in human training trials.

Because the reviewed studies differ in how closely their populations correspond to HHD, evidence is labeled by class throughout. HHD-specific direct evidence requires defined hypertension-mediated cardiac injury. Hypertension-specific supportive evidence comes from hypertensive cohorts not selected for cardiac injury and carries the blood-pressure, autonomic, vascular, and prevention questions. Cohorts in which hypertension predominates without defining etiology, including most HFpEF trials, are treated as predominantly hypertensive mixed evidence. Remaining human cohorts are indirect evidence, and animal or molecular studies are preclinical evidence used for mechanism only. Non-hypertensive cardiomyopathies, valvular disease, and athlete’s-heart physiology appear only as diagnostic or mechanistic boundaries. No training-effect trial reviewed here meets the HHD-specific direct standard, which is itself one of the principal findings. The prognostic and stress-imaging studies in defined hypertensive cardiac injury do meet it, but they test exposure or measurement rather than an intervention.

Trial-level effects and confidence intervals are reproduced from the cited reports or published syntheses without recalculation. Pooled estimates quoted in the text are those published by the syntheses cited; we generated no new pooled estimate and no heterogeneity statistic. Supplementary Materials Table S1 records design, population, co-intervention, adherence, attrition, outcome, and reporting limitations that materially affect interpretation. The table is not a domain-level RoB 2 assessment, a TESTEX score, or a formal certainty grade. Study selection followed relevance to the clinical questions and evidence classes set out above rather than a PRISMA-style exhaustive process, and no protocol was registered in advance. The retained search records are supplied to make that narrative selection transparent. Supplementary Materials Table S1 records the human training studies whose design or reporting limitations most affect how the review’s structural, vascular, autonomic, and functional conclusions should be read. It is a selection rather than a census of the cited studies, and preclinical, cross-sectional, and purely observational sources are not tabulated.

## 3. Pathophysiological Framework

In hypertensive heart disease (HHD), chronic pressure overload from arterial hypertension propagates through coupled neurohormonal, vascular, and myocardial mechanisms. The overload is the proximal insult: the left ventricle accommodates the sustained load first by concentric left ventricular hypertrophy (LVH), then by reactive interstitial and perivascular fibrosis. The fibrotic response is an excess of cross-linked type I collagen, which stiffens the wall, raises diastolic filling pressures, and can persist even after blood pressure is controlled. In a proportion of patients, the sequence culminates in overt hypertensive heart failure with preserved ejection fraction (HFpEF), which is one possible expression of HHD rather than an inevitable end stage [5,6,19,20]. Endothelial dysfunction and large-artery stiffening supply much of the afterload that perpetuates the overload, while sympathetic overactivity and an engaged renin–angiotensin–aldosterone system (RAAS) act upstream as shared drivers of both myocardial and vascular injury [16,21,22]. The mechanisms respond on different timescales and are gauged by markers of unequal sensitivity, so the effects of treatment are not always mirrored by resting imaging. Mechanism and clinical readout must therefore be read side-by-side [18].

Clinically, HHD is better handled as a graded phenotype than as a single diagnosis, and progression through it is neither obligatory nor uniform. Hypertension without detectable cardiac involvement is a precursor state rather than established HHD. Beyond it, three stages can be separated: concentric remodeling or LVH with preserved diastolic function; LVH with diastolic dysfunction but no symptoms; and symptomatic hypertensive HFpEF, with a minority progressing to reduced ejection fraction [5,19,20]. The stages differ both in what exercise can plausibly achieve and in how much evidence exists. Trials in uncomplicated or resistant hypertension address the earliest stages, small structural trials address the second, and mixed-etiology HFpEF trials address the last. The available evidence is therefore distributed unevenly along the continuum the disease itself follows.

The prognostic case for reversing the remodeling is strong. LVH affects roughly 36–41% of hypertensive patients, over half of those at high risk, and independently doubles all-cause mortality (relative risk 2.13, 95% CI 1.89–2.40). LVH is nonetheless modifiable: lowering blood pressure regresses LV mass, and normalizing an abnormal geometry improves survival (relative risk 0.64, 95% CI 0.42–0.97) [4]. Because pressure-overload hypertrophy follows calcium- and RAAS-dependent programs distinct from physiological growth, cardiac mass itself is not the therapeutic target. The question is whether the afterload-driven, maladaptive component of remodeling can be attenuated, given that exercise improves cardiac performance and vascular biology even when resting structural indices move little [21,23].

## 4. Mechanisms and Clinical Evidence

Exercise may act on the hypertensive heart at several steps of the continuum, and each pathophysiological claim below is set against the human evidence that tests it. The account is ordered from upstream drive to downstream endpoint. Representative studies are summarized against their evidence class in Table 1. The design and reporting limitations of the key human studies are set out in Supplementary Materials Table S1.

### 4.1. Autonomic and Neurohormonal Drive

Sympathetic overactivity is a defining, early feature of the hypertensive state and one of the most upstream contributors to hypertensive heart disease (HHD) [15]. Prospective data place the neural lesion ahead of overt hypertension. In the ELSA-Brasil cohort of 7665 normotensive adults, low resting heart rate variability (HRV) predicted incident hypertension over four years, with an adjusted relative risk of 1.79 (95% CI 1.36–2.35) for reduced low-frequency power. A comparable excess risk persisted in participants whose baseline pressure was normal [30]. Because depressed HRV indexes blunted vagal modulation and relative sympathetic predominance, autonomic impairment precedes the hypertension from which HHD develops. No cohort has tested whether the same holds for the cardiac injury itself.

The autonomic lesion is a disorder of reflex control: arterial baroreflex sensitivity (BRS) is depressed and reset upward while chemoreflex and exercise-pressor-reflex gain rise. Together they chronically disinhibit a central sympathetic outflow tightly coupled to the renin–angiotensin–aldosterone system (RAAS), since sympathetic traffic stimulates renin release and central angiotensin II excites sympathoexcitatory neurons while suppressing vagal outflow [3,15,16]. Conditioning alone does not erase the lesion. Amateur runners with untreated stage-1 hypertension, trained for 8–10 years, had 24% higher muscle sympathetic nerve activity (MSNA) than normotensive runners. Baroreflex control of MSNA and carotid elastic properties did not differ between the groups [31]. The comparison is cross-sectional and has no pre-training measurement, so it identifies a residual sympathoexcitatory substrate in trained hypertensive individuals rather than the extent to which training reduced it.

In established hypertension, structured training attenuates but does not normalize each component of the dysregulation [16]. Laterza and colleagues randomized never-treated essential hypertensives to four months of aerobic training or no exercise. Training restored baroreflex control of MSNA to normotensive levels, lowered MSNA from 35 ± 1 bursts/min toward the normotensive ∼22, and reduced clinic pressure from 145/94 to 130/84 mmHg [32]. Across trained and untrained hypertensive patients pooled, the change in MSNA correlated with the change in mean pressure (r=0.62, p<0.01), but the association weakened when only the trained patients were considered. The authors judged their data insufficient to support a causal link. Other modalities show the same. Home isometric wall-squat training raised BRS by 47% and cut 24 h ambulatory pressure by 11.8/5.6 mmHg [33], evening aerobic training lowered MSNA and pressure in treated elderly hypertensives [34], and resistance work tended to raise vagal HRV indices [35]. Combined aerobic–resistance rehabilitation cut ambulatory blood-pressure variability beyond the fall in mean pressure, which aerobic training alone did not [36]. For cardiac autonomic outcomes specifically, modality is not fully interchangeable: resistance-dominant programs can lower pressure while leaving cardiac autonomic indices unchanged or directionally adverse [37]. The chronic adaptation is sympathoinhibitory. Regular training cuts resting sympathetic traffic by close to 40% across microneurography studies and restores functional sympatholysis, so exercising muscle can override α-adrenergic vasoconstriction, unlike the transient excitation of a single bout [16,38].

The autonomic adaptation matters because it acts on a substrate that pressure control does not directly target. In 16 hypertensive men established on losartan, adding sixteen weeks of aerobic training raised baroreflex gain from 5.5 to 8.0 ms/mmHg and shifted HRV toward vagal dominance [39]. Both study groups trained and there was no drug-only comparison arm. The finding is therefore a before-and-after observation in treated patients rather than evidence that pharmacotherapy fails to modify autonomic disease. Training lowers ambulatory pressure additively to RAAS inhibitors without further suppressing renin or aldosterone [40]. Because sympathetic overactivity itself drives hypertensive target-organ damage, the reduction in sympathetic drive carries a prognostic rationale beyond the pressure effect [3].

Whether the autonomic adaptation drives the downstream structural and pressure benefit remains unproven: the change appears to be an early signal rather than an established mediator. HIIT lowered MSNA similarly in hypertensive and normotensive men (−17% vs. −27%) yet lowered pressure only in the hypertensive group, with no individual-level correlation between the two changes [41]. In TRIUMPH, four months of rehabilitation improved BRS (+2.3 vs. −1.1 ms/mmHg, p=0.003) and lowered 24 h systolic pressure by 7 mmHg, yet pulse-wave velocity and left ventricular mass held unchanged [42]. Early sympathoinhibition and structural reversal thus dissociated. The same dissociation extends to heart-rate control in hypertensive HFpEF. Chronotropic incompetence affects roughly half of patients and is associated with a lower peak VO_2_, yet neither high-intensity interval nor moderate training improved chronotropic reserve even as capacity rose. A second autonomic index therefore fails to explain the gain in capacity [43]. Measurement compounds the uncertainty: resting short-term HRV is often insensitive to training-induced change [44,45,46], aerobic training can lower ambulatory pressure without reducing blood-pressure variability [47], and MSNA microneurography stays confined to specialist centres. The size of the pressure reduction is also dose-dependent, since higher intensities cut non-response from 67% to 27% [48]. The autonomic evidence is therefore supportive rather than HHD-specific. Most of it comes from hypertensive cohorts not selected for cardiac injury; the HFpEF and rehabilitation cohorts drawn on above are predominantly hypertensive mixed evidence, and one is largely a coronary population. On either reading, it establishes an autonomic response to training rather than a mechanism of cardiac reverse remodeling. The upstream drive reaches the rest of the continuum through two routes, systemic vascular afterload and a direct, load-independent action on the myocardium.

### 4.2. Endothelial Function and Arterial Stiffness

At the vessel wall, the neurohormonal drive becomes mechanical afterload and feeds a self-reinforcing loop between rising pressure and arterial stiffening [15]. Two coupled lesions define the hypertensive vessel. Renin–angiotensin–aldosterone system (RAAS) activation uncouples endothelial nitric oxide synthase from nitric oxide (NO) and impairs endothelium-dependent vasodilation, measured as flow-mediated dilation (FMD) [22]. Separately, elastin fragmentation and medial calcification raise pulse wave velocity (PWV), particularly in the carotid–femoral segment (cfPWV), and increase central aortic load. The two lesions are gauged by endpoints that are not interchangeable. FMD and reactive hyperemia report conduit and microvascular endothelial function in one limb. Augmentation index and central blood pressure report the pulsatile load reaching the ventricle, and carotid–radial and carotid–femoral PWV report the regional stiffness of muscular and elastic arteries, respectively. Improvement in one endpoint is therefore treated below as evidence about the compartment measured, and any inference from one compartment to another is identified as a hypothesis rather than a finding. That the arterial lesion is nonetheless pressure-driven is shown over a 36-year cohort. The highest quartile of time in target range for systolic BP carried lower midlife brachial–ankle pulse wave velocity, independent of mean pressure and blood-pressure variability [49].

Blood-pressure lowering is the most proximal vascular effect of training and is clearest on ambulatory BP. The effect holds even where pharmacotherapy is exhausted: in resistant hypertension on optimized therapy, EnRicH cut 24 h ambulatory systolic BP by 7.1 mmHg with 12 weeks of aerobic training, comparable to renal denervation [9]. Walking added to four-drug therapy lowered ambulatory BP further [50]. The effect is modality-tolerant [22] yet modest at scale (about 1 mmHg across 31 885 cardiac-rehabilitation patients [51]). Time-efficient protocols can match longer ones: in healthy community-dwelling older adults a 15 min isometric handgrip protocol and a high-intensity interval protocol each lowered systolic BP by 9 mmHg [52]. Isometric work beat aerobic training for central and ambulatory diastolic BP in hypertensive participants [53]. Judging the benefit from office readings alone overstates the true hemodynamic gain, and the discrepancy recurs across modalities. Aerobic training lowered daytime ambulatory BP but not short-term variability, an independent risk marker [47] that combined training reduced more than aerobic did [36]. Resistance training moved neither 24 h BP nor variability despite an office fall [54], home handgrip left 24 h BP unchanged despite an 8 mmHg office fall [55]. Single bouts left 24 h BP, FMD, and heart-rate variability untouched [56].

Beyond blood pressure, endothelial and microvascular endpoints improve earlier and more consistently than conduit stiffness. Aerobic, resistance, or combined training each raised brachial FMD by 3.2–6.8% (p≤0.006), yet 24 h systolic BP fell only in the aerobic and resistance arms [24]. Resistance training lowered central systolic BP by 6.8 mmHg and improved FMD in proportion to the peripheral systolic BP reduction while cfPWV was unchanged [57]. Dynamic resistance training raised microvascular reactive-hyperemia flow while FMD again stayed unchanged [58]. Six weeks of passive lower-limb stretching in hypertensive individuals separated the compartments more sharply still, lowering systolic BP by 4.9% and diastolic BP by 6.9% and raising peak femoral flow by 18.4%. Brachial FMD, nitrate-mediated dilation, and cfPWV all stayed unchanged [25]. Every gain was lost during follow-up. Inspiratory muscle training raised FMD by about 45% alongside increased NO bioavailability, again with cfPWV unchanged [59]. The NO axis is a plausible common mechanism but cannot be the only one, since neither HIIT nor moderate training shifted circulating NO-pathway metabolites in predominantly hypertensive HFpEF [60].

Conduit-artery stiffness is the less tractable target, and it responds only to a sufficient training dose. Interpretation is further constrained by measurement. Carotid–femoral PWV rises with the prevailing distending pressure. Trials that lower mean arterial pressure and report an unadjusted fall in cfPWV therefore cannot separate intrinsic arterial destiffening from the mechanical consequence of a lower operating pressure. One hypertensive resistance-training trial added the change in mean arterial pressure as a covariate for precisely that reason [57]. The converse also holds, since an unchanged unadjusted cfPWV does not exclude a change in intrinsic wall properties masked by a lower distending pressure. The term destiffening is therefore not applied to an arterial endpoint below unless the design separates the two. Rigorous trials lowered BP with no change in large-artery stiffness: aerobic training in resistant hypertension left arterial compliance unchanged [50]. The EnRicH secondary analysis cut central systolic BP by 12.2 mmHg and circulating angiotensin II while cfPWV stayed flat [61]. The dissociation is compatible with RAAS-mediated or microcirculatory rather than aortic mechanisms, although neither compartment was measured. The segment measured matters, though not in a consistent direction: training cut peripheral muscular-artery PWV by roughly 1 m/s while central elastic-artery PWV resisted change [62]. Handgrip training, by contrast, reduced central but not peripheral PWV [63]. Established remodeling also blunts the reduction in hypertensive versus normotensive women [64]. Adequate dose nonetheless moves conduit stiffness. ENCORE added aerobic training and weight loss to the Dietary Approaches to Stop Hypertension (DASH) diet and produced greater reductions in PWV and LV mass, and greater improvement in baroreflex sensitivity, than diet alone [65]. Because the active arm combined three components, the effect cannot be attributed to training alone. Power training plus walking reduced PWV by 0.3 m/s [66]. Pooled across fourteen randomized trials in hypertension, exercise reduced pulse wave velocity by 0.76 m/s (95% CI 0.47–1.05). The reduction was largest for isometric work (0.98 m/s) and not significant for dynamic resistance training (−0.58 m/s, 95% CI −1.58 to 0.42) [26]. Because the pooled trials report PWV without uniform pressure adjustment, the estimate quantifies the change patients experience rather than the change in intrinsic wall stiffness.

What ultimately matters is the load the ventricle actually meets. Ventricle and arterial tree operate as a coupled system, and stiffening raises central pulsatile load as well as mean afterload. Antihypertensive therapy improves ventricular–arterial coupling, lowers cardiac work, and improves LV efficiency, with attenuated responses in women and in obese hypertensive patients [20]. Coupling also constrains what the hypertensive heart can do under load. In treated hypertensive patients with exertional dyspnea, the blunted rise in stroke volume at peak exercise has been attributed to abnormal ventricular–arterial coupling, which may in turn drive progression of diastolic dysfunction [67]. Coupling is quantified as the ratio of effective arterial to end-systolic elastance. The arterial waveform used to derive the ratio reflects ventricular ejection as well as arterial properties, so neither limb can be read as a pure measure of the other. Training may act on the coupling from the arterial side, since central systolic BP fell by 6.8 and 12.2 mmHg in two hypertensive trials whose cfPWV did not change [57,61]. No trial in a cohort defined by hypertension has reported a coupling index before and after training, so the interpretation remains a hypothesis. A fall in central pressure and a fall in conduit stiffness are in any case distinct results.

The blood-pressure benefit therefore often arrives without any demonstrated change in intrinsic conduit stiffness. Falling sympathetic drive and restored functional sympatholysis are plausible contributors [38], although no hypertensive training trial has tested the mediation directly. The vascular evidence is likewise supportive rather than HHD-specific. Most trials enrolled hypertensive adults not selected for cardiac injury. The section additionally draws on a healthy older cohort, a mixed cardiac-rehabilitation registry, and a predominantly hypertensive HFpEF trial, each of which is further still from established HHD. The evidence is accordingly more consistent for endothelial and microvascular function than for reversal of fixed conduit damage.

### 4.3. Left Ventricular Remodeling

**Figure 1:**
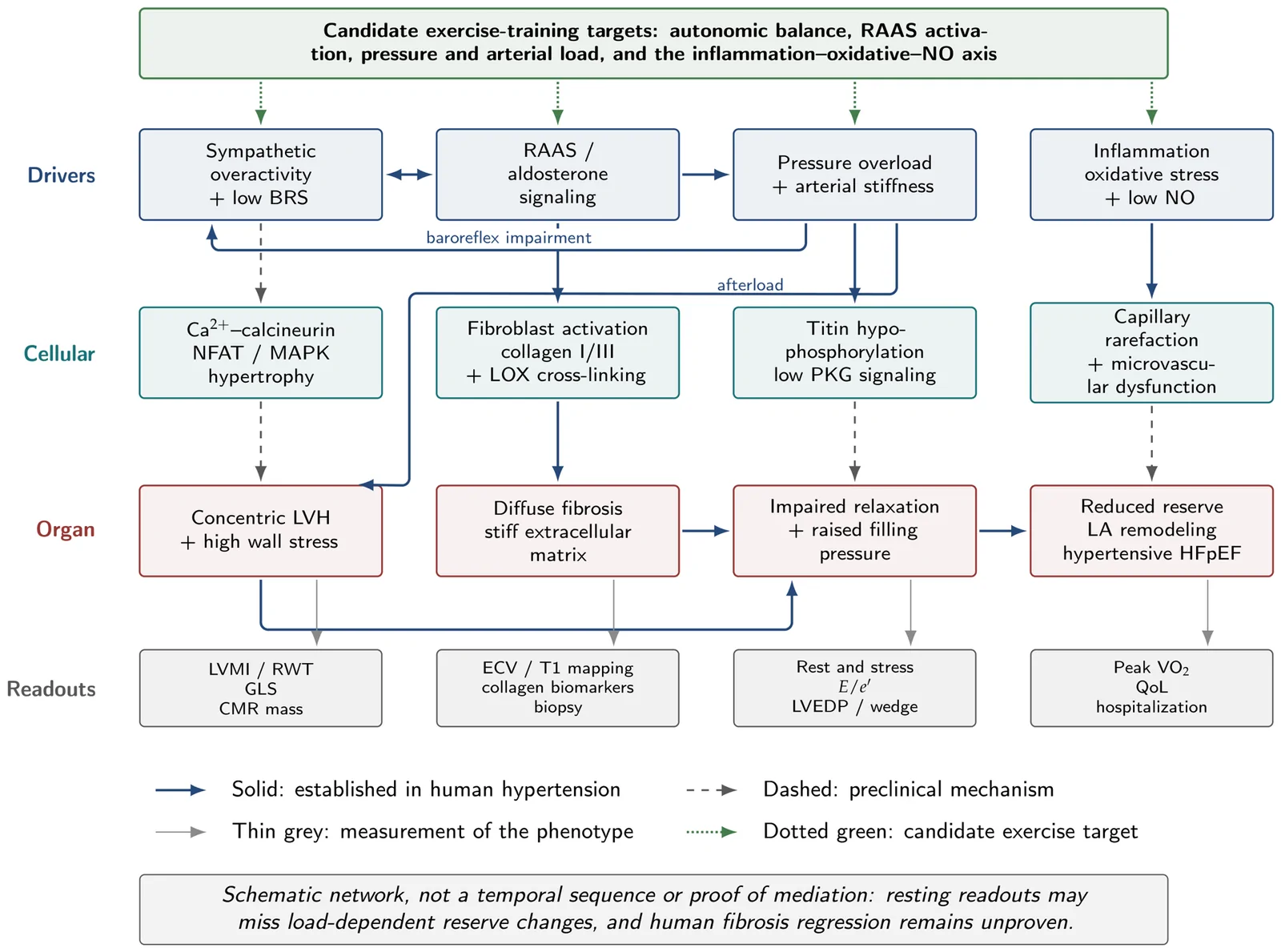
Mechanistic network of HHD and candidate exercise-training targets. The diagram separates upstream drivers, cellular pathways, organ-level phenotypes, and the readouts used to measure them, and codes each link by the strength of the evidence behind it. The eight vertical links between bands are coded on their own evidence rather than on the bands they join, so line style varies within and between columns; the horizontal links, the measurement links, and the exercise-target links each carry one style by kind. Solid arrows mark relationships for which human hypertensive evidence is cited in the corresponding section: the coupling between sympathetic drive and RAAS activation, the feed-forward limb through which arterial stiffening impairs the baroreflex and re-excites sympathetic outflow, the afterload route from pressure overload to concentric hypertrophy, the convergence of hypertrophy and fibrosis on impaired relaxation and then on hypertensive HFpEF, and the vertical steps documented in human myocardium or in patients, namely, RAAS-driven fibroblast activation and the collagen it deposits, titin hypophosphorylation, and microvascular dysfunction. Dashed arrows mark steps resting mainly on preclinical models, namely, calcineurin-NFAT hypertrophy signalling and its organ-level consequence, the step from titin to impaired relaxation, and the step from capillary rarefaction to reduced reserve. Thin grey arrows are measurement links rather than evidence claims, and the four dotted green arrows mark the upstream drivers that exercise training is proposed to act on. The horizontal solid arrows carry the coupling that makes the diagram a network rather than four parallel chains. The network is neither a fixed temporal sequence nor proof of exercise-mediated causation.

Under sustained vascular afterload, compounded by the direct, load-independent myocardial action of sympathetic and renin–angiotensin–aldosterone system (RAAS) drive, the left ventricle responds with concentric hypertrophy and interstitial fibrosis. The remodeling is largely maladaptive, and whether exercise training can attenuate or reverse it is the central question; reversal in HHD targets the afterload-driven component, the molecular-to-organ cascade of Figure 1.

The afterload-driven remodeling has a defined molecular signature, and pressure-overload animal models show exercise engaging parts of it, though not always in one direction. Calcineurin–NFAT and mitogen-activated protein kinase (MAPK) signaling dominate pathological hypertrophy, while pressure overload and RAAS activation drive fibroblast-to-myofibroblast conversion, excess collagen I and III deposition, and capillary rarefaction, leaving a stiff, hypoperfused substrate [21]. In aged spontaneously hypertensive rats (SHR), eight weeks of high-intensity interval training lowered systolic BP (229 to 199 mmHg, p=0.001), quadrupled exercise tolerance, and restored the MAPK transcription factor ATF2 toward normal (p=0.0002) [68]. The same protocol also increased indices of cardiac hypertrophy, which the authors report did not worsen systolic or diastolic function. Moderate treadmill training in ovariectomized SHR downregulated angiotensin II type-1 receptor expression and the TGF-β and NF-κB fibrotic cascades [69]. In transverse aortic constriction, aerobic exercise and swim preconditioning curbed hypertrophy and collagen through mitophagy, inflammasome, and SIRT1–NRF2 signaling [70,71], and an exercise-released peptide attenuated angiotensin II–induced hypertrophy and fibrosis [72]. Resistance exercise reproduces the signature in SHR only with adjunctive antioxidant supplementation [73]. Epigenetic regulation has been proposed as a further layer. In rodents subjected to transverse aortic constriction, presented as an experimental model of heart failure caused by chronic arterial hypertension, exercise modulates non-coding RNAs, histone modification, and DNA and RNA methylation [74]. The evidence is preclinical, so the pathway remains a candidate rather than an established route in patients. A systematic review of hypertensive-heart experiments nonetheless found only limited fibrosis-specific evidence, with two studies reporting improved fibrotic characteristics after training [75]. The animal work cannot fix the magnitude of human reversal, but it converges on one claim: exercise engages the RAAS, MAPK, redox, mitochondrial, and matrix nodes through which hypertension produces fibrosis and concentric growth.

In hypertensive patients, the human evidence for reverse remodeling is strongest for geometry and weakest for mass. A controlled trial assigned overweight, sedentary adults with high-normal or mildly elevated BP to six months of aerobic exercise, exercise plus weight management, or control. Analysis was confined to the 82 of 144 participants with interpretable paired echocardiograms, five of whom were non-randomized controls. Relative wall thickness (RWT) fell 6–7% in the active groups (p=0.003), with thinner septal (p=0.004) and posterior (p=0.05) walls, whereas the left ventricular mass index (LVMI) showed only a non-significant trend (p=0.08) partly offset by modest chamber enlargement [27]. A head-to-head comparison against pharmacotherapy shows both the efficacy and the limit of exercise. In older adults with mild hypertension, six months of endurance training cut LVMI by 14% and the wall thickness-to-radius ratio (0.48 to 0.42), matching hydrochlorothiazide despite only about half the systolic BP fall. Roughly half the exercise arm withdrew, however, and the analysis was not by intention to treat. Only training added a 15% VO_2_peak rise [28]. The fall in the wall thickness-to-radius ratio tracked the individual systolic BP fall in the exercise arm (r=0.70, p=0.005) but not in the diuretic arm. Yet, afterload reduction and structural reversal are not interchangeable: BP-lowering magnitude does not reliably predict LVH regression [5], which points to the direct, load-independent cellular effects the animal data describe.

Several trials nonetheless pair robust BP effects with structural endpoints that remain trends. A 16-week multimodal telerehabilitation program in medicated hypertensive older adults lowered office systolic BP by 11.4 mmHg but produced only non-significant reductions in RWT and LVMI. The authors suggest the sixteen-week stimulus may have been insufficient [76]. A one-year lifestyle trial in type 2 diabetic adults, HHD-adjacent rather than pure-hypertensive, halved progression to a hypertensive exercise response (29.8% vs. 59.5%, p=0.006) yet moved neither LVMI nor RWT [77]. Apparent failure to regress should first prompt review of office and ambulatory blood pressure, treatment adherence, the duration of follow-up, and the reproducibility of the measurement itself, since each can produce a spurious non-regressor. Only then should the diagnosis be reconsidered. Hypertrophic cardiomyopathy, cardiac amyloidosis, Fabry disease, and valvular disease all mimic hypertensive LVH and do not respond to afterload reduction. Persistent hypertrophy in a treated patient therefore warrants multimodality imaging and, where indicated, genetic or tissue confirmation before it is attributed to residual hypertensive load [5,20].

Whether the weak mass and fibrosis signal reflects genuine resistance to reversal or the insensitivity of resting linear indices to slow interstitial change remains unresolved. Tissue characterization could close the detection gap. Cardiac magnetic resonance T1 mapping and extracellular volume (ECV) quantify the diffuse interstitial fibrosis defining the hypertensive substrate, present on ECV mapping in more than 40% of preserved-ejection-fraction hearts and on biopsy in over 90% [5]. Global longitudinal strain (GLS) exposes subclinical contractile loss before ejection fraction falls. We found no hypertensive-exercise trial reporting serial ECV or biopsy-proven fibrosis regression. The antifibrotic signal therefore rests on animal work, and even there, a systematic review of hypertensive-heart experiments found fibrosis-specific evidence limited [75].

Beyond whether reversal occurs, the available modality comparisons are reassuring rather than definitive, and each rests on a small and narrowly selected cohort. Swim training and recreational soccer in mildly hypertensive women both raised LVMI alongside improved filling and concurrent BP reduction, an adaptive rather than adverse mass gain [78,79]. Isometric training is too brief to induce volume-loaded mass change. Four weeks of unsupervised home-based wall-squat exercise in stage-1 hypertensives nonetheless lowered resting systolic BP by 12.4 mmHg, improved GLS by 2.3%, and reduced estimated filling pressure ($E/e^{\prime }$ −1.1, p=0.001). The gains reversed within three weeks of stopping [80].

Population cohorts link habitual physical activity to lower incident LVH and lower mortality. In the HARVEST cohort of young, untreated stage-1 hypertensives, regular physical activity over a median 8.3 years was associated with lower incident LVH (10.3% to 1.7%; adjusted odds ratio 0.24, 95% CI 0.07–0.85). The association persisted after adjustment for the achieved BP change [81]. Activity was self-selected and BP change is a mediator rather than a confounder, so the analysis is compatible with, but does not establish, a BP-independent pathway. The inverse physical-activity–LVMI relationship is steeper in hypertensives than normotensives [82], and higher activity predicted lower mortality even among hypertensives with established LVH (hazard ratio 0.61, 95% CI 0.38–0.97) [83]. The LIFE echocardiographic substudy of 937 hypertensive patients with electrocardiographic LVH makes the same point: baseline physical activity carried 58% lower composite-endpoint risk (hazard ratio 0.42, 95% CI 0.26–0.68) [84]. The benefit nonetheless distributes unevenly across sex, age, and metabolic comorbidity. The activity–LVMI relationship is conditioned by adiposity as well as sex [82], the favorable adaptation to soccer and swimming appeared in premenopausal women [78,79], and the response blunts with age [76].

One caveat tempers the structural evidence: an early hyperkinetic phase narrows the reversal window. In a cross-sectional comparison with matched normotensive controls, newly diagnosed untreated hypertensives already showed higher LVMI (105.6 vs. 93.4 g/m^2^, p<0.001) and greater arterial stiffness while diastolic function was still normal [85]. Whether the window for reversal narrows as fibrosis accrues is a plausible reading of that sequence rather than something the design can test.

Across animal mechanism, controlled trials, and population cohorts, exercise opposes hypertensive remodeling chiefly by unloading afterload. The structural evidence is, however, thinner than the vascular and autonomic evidence. The human trials are small, enrolled mild or high-normal rather than established HHD, and used resting linear indices, while the antifibrotic case rests on preclinical models. Within the one exercise trial that tested it, geometric regression tracked the individual BP fall [28]. Across drug classes, by contrast, the magnitude of BP lowering does not predict the magnitude of LVH regression [5]. The two observations describe different comparisons rather than a single dose–response relationship. Load-independent antifibrotic and prognostic signals remain plausible but incompletely proven in humans.

### 4.4. Diastolic Function and HFpEF

Pressure overload builds a stiff structural substrate with a predictable functional consequence: concentric left ventricular hypertrophy (LVH) and interstitial collagen I and III accumulation. Titin shifts toward a less compliant isoform and is hypophosphorylated once nitric oxide (NO)–cyclic GMP–protein kinase G signaling falls. The stiffened ventricle then fills only at the cost of elevated pressure. Left ventricular end-diastolic pressure (LVEDP), the ratio of early mitral inflow to mitral annular velocity ($E/e^{\prime }$), and left atrial pressure all rise, first on exertion and later at rest [5,19,20].

The progression from hypertensive remodeling to heart failure with preserved ejection fraction (HFpEF) defines the phenotype examined here [19,20]: HFpEF arising from hypertension rather than from obesity, ageing, or the hypertrophic phenocopies. The hypertrophic phenocopies must be excluded before exercise becomes a remedy. Hypertension is the comorbidity most strongly tied to the defining LVH and fibrosis, yet the link is probabilistic rather than obligate. Concentric remodeling or hypertrophy is present in roughly half of HFpEF cohorts and independently predicts heart-failure hospitalization and cardiovascular death (LVH hazard ratio 1.86, 95% CI 1.40–2.46 in I-PRESERVE). Fibrosis on extracellular-volume mapping carries a hazard ratio of 1.75 per 5% increment (95% CI 1.25–2.45) [5].

Animal models isolating the hypertensive insult reproduce the cascade. Angiotensin II and mineralocorticoid models both generate concentric hypertrophy, elevated LVEDP, and fibrosis with preserved ejection fraction [86,87]. The lesion is fibrotic as much as hypertrophic: preventing CCR2-dependent macrophage recruitment in a genetic loss-of-function model limited hypertrophy but not fibrosis or diastolic dysfunction [88]. In an aged-ewe renovascular preparation, conscious resting cardiac output stays near-normal while directly measured LVEDP is already elevated (13 ± 5 vs. 0.5 ± 1 mmHg, $p\approx 2\times 10^{-6}$). Treadmill exercise then unmasks a blunted cardiac-output reserve and an augmented wedge-pressure rise (p=0.026) [89]. Cited only for mechanism, the models converge: the hypertensive diastolic lesion is masked at rest and emerges only under load.

For humans, the effect on exercise capacity is consistent and the effect on resting diastolic function is not. Table 2 sets the individual trials and the pooled syntheses side-by-side. The encouraging early signal from the Ex-DHF pilot [90] narrowed as evidence accumulated. Fitness and quality of life improved across successive syntheses while ejection fraction, natriuretic peptides, and E/A ratio did not. The one synthesis reporting a fall in resting $E/e^{\prime }$ was not reproduced by the 2024 update [23,29,91]. The main Ex-DHF trial confirms the dissociation at scale. In 322 patients, 84% hypertensive, the hierarchical primary endpoint went unmet while peak VO_2_ and New York Heart Association (NYHA) class improved and septal $E/e^{\prime }$ and clinical events did not [92]. Higher intensity does not rescue the resting endpoint, and by twelve months the OptimEx-Clin between-group differences had dissolved as home-based adherence fell [93]. Its exercise-echocardiography substudy made the point resting imaging cannot: capacity rose alongside a small but significant between-group improvement in maximal-exercise $E/e^{\prime }$. The two changes were nonetheless unrelated, so better diastolic function does not explain the capacity benefit [94]. The pooled cohorts are phenotype-mixed, so the magnitude is generic rather than hypertension-specific [23].

Two explanations reconcile functional gain with diastolic stasis, and both implicate resting echocardiography. First, adaptation occurs largely outside the heart, in skeletal-muscle oxidative capacity, oxygen extraction, and vascular function, and raises peak VO_2_ without changing ventricular filling [92,94,95]. Second, resting indices cannot detect an abnormality that appears only under load. Across eighteen HFpEF training trials, resting $E/e^{\prime }$ improved in only five of twelve studies and left atrial volume index in three of seven [18]. Stress imaging confirms the abnormality is real but exertional. In treated, uncomplicated hypertensives with exertional dyspnea but normal resting echocardiography, exercise impedance cardiography exposed a blunted cardiac-output reserve (peak 13.1 vs. 17.0 L/min, p=0.002) and shorter six-minute walk distance [67]. Exercise $E/e^{\prime }$ rather than the resting value predicted coronary microvascular dysfunction in untreated hypertensives [96], and hypertensive controls matched on resting $E/e^{\prime }$ escaped the exercise-induced diastolic ventricular interaction that defined HFpEF [97]. The atrium behaves the same way: in 139 strictly hypertensive patients, exercise left-atrial reservoir-strain reserve fell progressively across heart-failure stages (8.4% to 1.2%, A vs. C, p<0.001). The reserve also predicted incident heart failure or atrial fibrillation incremental to resting indices [98]. Trials gauging efficacy by resting $E/e^{\prime }$, LVMI, or natriuretic peptides therefore incompletely capture the exercisable lesion.

Dose and modality remain unsettled, and whether the hypertensive phenotype needs a tailored prescription is explicitly unresolved in a dedicated position statement on exercise in hypertensive HFpEF [17]. Randomized comparison found no significant difference between HIIT and moderate continuous training for capacity, which is not the same as demonstrated equivalence [23,93]. A mechanistic substudy nonetheless reports an endothelial nitric-oxide signal after HIIT but not after moderate training, which its authors attribute to higher shear forces that were not measured [95]. Isometric exercise has now entered HFpEF testing: a 38-patient, four-week randomized study lowered resting systolic pressure by 8.5 mmHg and improved global longitudinal and left atrial reservoir strain. The trial was powered for blood pressure and explicitly requires larger multicentre confirmation [99]. Effects attenuated once supervision ended in both OptimEx and its mechanistic substudy, which implicates adherence, although neither trial randomized supervised against home-based delivery [93,95]. The hard-endpoint evidence is no longer empty but remains non-definitive for HHD, since the one event meta-analysis reports lower rehospitalization without a mortality reduction, again in phenotype-mixed cohorts [100]. Prevention offers the one population-scale signal upstream of symptomatic HFpEF. In a Women’s Health Initiative cohort of 137,303 postmenopausal women, with heart-failure subtype adjudicated in a subcohort, each one-log increment of physical activity carried roughly 8% lower HFpEF incidence. The fully adjusted trend across activity tertiles nonetheless did not reach significance [101]. Observational and mixed in etiology, the last finding is compatible with, but does not establish, an association that begins before remodeling is established.

**Figure 2:**
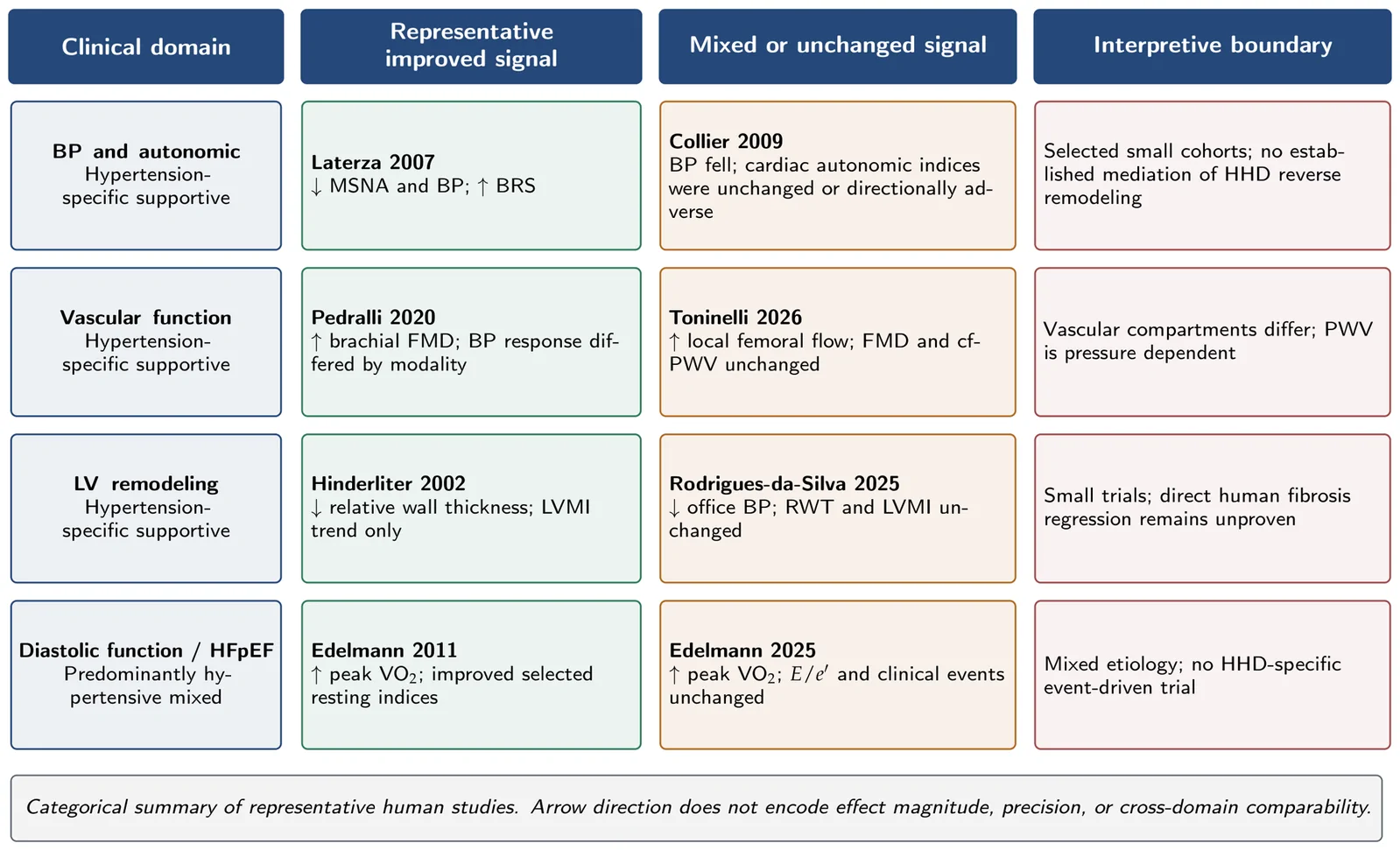
Domain-specific matrix of representative human exercise-training studies. Each row separates an improved signal from a mixed or unchanged signal and states the principal interpretive boundary. The display is categorical: arrows indicate direction only and do not place unlike outcomes on a common effect-size scale. Representative studies are discussed and cited in the corresponding sections [24,25,27,32,37,76,90,92].

Across the continuum, exercise behaves as a mechanism-targeted adjunct that engages neurohormonal, endothelial, and reserve substrates in addition to those reached by antihypertensive drugs. The HFpEF evidence is the clearest case of predominantly hypertensive mixed evidence. The flagship trials are 84–86% hypertensive, and up to 93% in one substudy, but none was restricted by etiology, so their estimates apply to HHD by inference rather than by design. The constraint is tempo: functional and peripheral benefit appears early, whereas mass regression, fibrosis regression, and resting diastolic reversal are inconsistently demonstrated, and event reduction in HHD is not established. An effective prescription therefore depends on knowing where benefit is large and rapid, where it is partial and delayed, and where load-based measurement is required to detect it at all. Representative studies are arranged by clinical domain in Figure 2.

**Clinical take-home points**

- Blood-pressure reduction and improved functional capacity are the most consistent human training effects in hypertension.
- Moderate aerobic training on 3–5 days per week, accumulated to 150–300 min/week, is the practical starting dose, with resistance and isometric work as additions and with the prescription sustained indefinitely, since benefit regresses on detraining.
- Autonomic, endothelial, and microvascular changes are measured responses rather than established mediators of cardiac remodeling, and vascular endpoints are compartment specific.
- Pulse-wave velocity varies with distending pressure, so a reduction alone does not establish intrinsic arterial destiffening.
- Small hypertension trials suggest that left ventricular geometry can improve, whereas human myocardial fibrosis regression remains unproven and resting diastolic indices change inconsistently.
- Exercise training adds to antihypertensive therapy and comprehensive risk-factor management and replaces neither; HHD-specific dose and clinical-event evidence remain limited.

**Table 2:** Exercise-training trials and syntheses in predominantly hypertensive HFpEF. Where a percentage is given, it is the proportion of the cohort with hypertension. No cohort was restricted by etiology, so the individual trials are predominantly hypertensive mixed evidence. The pooled syntheses and the event meta-analysis do not report the hypertension proportion and are therefore treated as indirect human evidence.

Study	Cohort and Intervention	Exercise Capacity	Resting Diastolic Index	Clinical Events
Ex-DHF pilot [90]	64 patients; 3 months supervised endurance-resistance	Peak VO_2_ +3.3 mL·kg^−1^·min^−1^	$E/e^{\prime }$−3.2 (95% CI −4.3 to −2.1); left atrial volume index lower	Not assessed
Ex-DHF main [92]	322 patients, 84% hypertensive; 12 months supervised combined	Peak VO_2_ +1.3 (0.4–2.1), p=0.003; NYHA class odds ratio 7.77 (3.73–16.21)	Septal $E/e^{\prime }$ −0.2 (−1.3 to 0.8), p=0.66	Death or cardiovascular hospitalization not different
OptimEx-Clin [93]	180 patients, 84–86% hypertensive; 3 months MCT or HIIT	MCT +2.0 (0.9–3.1); HIIT +1.5 (0.4–2.7); neither reached the 2.5 threshold; HIIT no better (p=0.41)	Medial $E/e^{\prime }$ and natriuretic peptides unchanged	Not powered for events; group differences gone at 12 months
OptimEx echo substudy [94]	61 patients, 93% hypertensive; exercise echocardiography	Peak VO_2_ +2.7 vs. +0.2, p=0.006	Maximal-exercise $E/e^{\prime }$ 18.3 → 17.2 vs. 21.7 → 22.8, p=0.044; changes uncorrelated	Not assessed
Sugita 2023 [102]	Older, lean, 68% hypertensive; 5 months combined, non-randomized	Peak VO_2_ and left atrial strain higher	$E/e^{\prime }$ and LV mass index unchanged	Not assessed
Pooled syntheses [23,29,91]	6, 11, and 14 randomized trials	Peak VO_2_ +2.33 (1.73–2.94) and +1.96 (1.25–2.68); quality of life improved	$E/e^{\prime }$−1.71 (p=0.005) in one synthesis; E/A, deceleration time and $E/e^{\prime }$ unchanged in the 2024 update	No trial reported hospitalization or mortality follow-up
Event meta-analysis [100]	19 randomized trials, 2025 participants	Not the endpoint	Not the endpoint	Rehospitalization risk ratio 0.52 (0.38–0.72); all-cause mortality 1.13 (0.77–1.66)

## 5. Exercise Prescription, Delivery, and Safety

### 5.1. Modalities and Dosing

Exercise in hypertensive heart disease is delivered as an individualized prescription alongside risk-factor management, with the training dose specified through the FITT framework of frequency, intensity, time, and type. Table 3 summarizes practical starting doses and the evidence boundary attached to each modality. A 2022 consensus document of the European Association of Preventive Cardiology and the European Society of Cardiology stratifies modality by baseline pressure, placing aerobic training first-line in established hypertension and using dynamic or isometric resistance work as adjunctive or selected alternatives according to risk and phenotype [8]. Moderate continuous aerobic training is the practical starting prescription, advanced gradually once resting and exercise blood pressure are controlled [8], and it lowers ambulatory pressure even in resistant hypertension when added to optimized therapy [9]. High-intensity interval training can be considered in stable, supervised patients who need a time-efficient stimulus, since it matches moderate training for pressure and aerobic capacity in a cardiometabolic population [11]. The comparison is indirect rather than hypertension-specific. An eight-week interval program nonetheless improved retinal microvascular but not conduit-artery function, so higher intensity does not reach every vascular bed equally [103].

Resistance and respiratory variants should be layered around that aerobic core rather than substituted indiscriminately. The value of dynamic resistance training is peripheral strength, vascular function, and long-term adherence rather than proven LVH regression [8,36,57]. A meta-analysis of 84 randomized trials in 5065 hypertensive patients nonetheless found dynamic and isometric resistance lowered blood pressure comparably to aerobic training, with no interaction by modality [104]. Resistance training is therefore a legitimate antihypertensive stimulus rather than only a supplement. Isometric handgrip or wall-squat training is the top-ranked modality for systolic and diastolic pressure in network and pairwise syntheses [10,12]. In established HHD, LVH, or HFpEF, it is best used as a selected adjunct with pre-session blood-pressure checks, since the supporting trials enrolled hypertensive adults rather than patients with cardiac injury [80]. The first small HFpEF randomized trial of isometric training supports feasibility and short-term blood-pressure and myocardial-mechanics signals, while leaving long-term safety, adherence, and event effects unresolved [99]. Inspiratory muscle strength training widens the same time-efficient niche, lowering systolic pressure by 9.1 mmHg in about eight minutes per session where thirty minutes of aerobic work lowered it by 6.2 mmHg. The two were not significantly different, and only the aerobic effect persisted after detraining [105]. Accumulated dose appears to matter more than weekly distribution. In accelerometer cohorts, a compressed weekend pattern was associated with a lower incidence of cardiovascular disease in hypertensive adults, to a degree similar to evenly distributed activity (hazard ratio 0.76, 95% CI 0.71–0.82 versus inactivity) [106]. Bout structure nonetheless appears to matter for vigorous work. In 38,960 adults with diagnosed hypertension, vigorous activity accumulated in short bouts was associated with lower major adverse cardiovascular events. The same intensity accumulated in bouts longer than two minutes was associated with a two-to-threefold higher stroke risk (hazard ratio 2.06, 95% CI 1.38–3.07, at 44 min/week). The risk rose with the weekly volume accrued that way (2.80, 1.72–4.56, at 64 min/week) [107]. Both cohorts are observational and the finding needs confirmation. Until the question is resolved, the safer working assumption is that vigorous work should be accumulated in short bouts.

### 5.2. Delivery and Adherence

Where exercise is delivered depends on the health system. Comprehensive cardiac rehabilitation is a multicomponent service in which supervised exercise sits alongside cardiovascular risk assessment, lipid and blood-pressure management, nutritional counselling, weight management, smoking cessation, psychosocial support, and long-term adherence strategies. The present review examines the exercise component rather than the full service. Rehabilitation programmes are generally organized around qualifying indications such as recent acute coronary syndrome, revascularization, or heart failure, so hypertension without a qualifying indication does not routinely enter a formal rehabilitation cycle in many systems. Exercise for HHD is therefore delivered mainly within routine hypertension care, through supervised rehabilitation services when a qualifying indication and local capacity exist, or through structured home and telehealth programs. In our own setting in China, the same constraint applies. Cardiac rehabilitation is organized around post-acute cardiac indications, so hypertension without cardiac injury is managed in outpatient hypertension and community primary-care clinics, and the exercise component is advice-based unless the patient qualifies for a programme. The practical consequence is common to both settings. Most patients with HHD will receive their exercise prescription from the clinician managing their blood pressure rather than from a rehabilitation service, which is why the dose in Table 3 is framed for that consultation. Consensus recommendations treat exercise as part of lifestyle management for treated hypertension and high-normal BP, and ask that available infrastructure be weighed alongside age, sex, comorbidity, and patient preference when a dose is set [8]. Exercise nonetheless remains significantly underused in hypertension, partly because of limited clinician knowledge and clinical inertia [8].

Supervised programs produce the largest and most reliable reductions, yet benefit can persist when supervision gives way to self-directed maintenance. Twelve supervised weeks of water- or land-based combined training followed by twelve self-supervised weeks lowered pressure under both formats in hypertensive older adults [108]. Home-based and digitally delivered rehabilitation extends reach where conventional programs enrol few. A smartphone-guided application lowered systolic pressure by 5.5 mmHg over standard care in uncontrolled hypertension [109]. An artificial-intelligence-guided telerehabilitation program improved six-minute walk distance by 62.8 m (95% CI 26.3–99.2) over eight weeks, against conventional exercise rehabilitation rather than usual care [110]. Adherence is the rate-limiting step. Supervised walking achieved 93% attendance across the exercise arms of a three-arm randomized trial in older hypertensive adults [111]. At the scale of real-world registries, however, the averaged pressure effect of rehabilitation is modest [51]. Whether benefit persists after training stops depends on the modality. Isometric wall-squat gains reversed within three weeks [80], and the inspiratory-training effect did not persist through four weeks of detraining although the aerobic effect did [105].

### 5.3. Safety and Phenotype-Guided Selection

Safety monitoring is integral to the prescription. Before escalation, clinicians should confirm resting blood-pressure control, recent medication changes, orthostatic symptoms, exertional dyspnea, angina, arrhythmia symptoms, and HFpEF congestion status. Perceived exertion, heart-rate response, symptoms, and exercise blood pressure should then guide progression, especially in patients with LVH, diabetes, kidney disease, or polypharmacy. An exaggerated exercise blood-pressure response does not by itself justify restricting exercise. Patients with high-risk LVH reach higher maximal exercise systolic BP (196 vs. 173 mmHg) and more often meet criteria for an exaggerated response (48% vs. 11%). The rise relative to absolute VO_2_ nonetheless matches healthy controls, so the finding largely reflects the higher resting pressure from which the response begins [7]. The appropriate reaction is assessment rather than prohibition: resting and ambulatory blood pressure, the workload-adjusted pressure response, hypertension-mediated organ damage, current medication, and cardiorespiratory fitness should each be reviewed. Uncontrolled resting hypertension should be treated before the training load is advanced.

Response is heterogeneous, with only about half of patients reaching a clinically important pressure reduction in some trials [112]. Prescription is accordingly moving from generic activity advice toward phenotype-guided selection, with modality matched to baseline pressure and to the dominant lesion [8]. The limits of what exercise substitutes for should be equally explicit. Improvement in flow-mediated dilation, pulse-wave velocity, blood pressure, or exercise capacity does not demonstrate coronary plaque regression, and it does not replace evidence-based lipid-lowering, glycemic, renal, weight-management, or smoking-cessation treatment. Exercise training is an addition to comprehensive cardiovascular risk-factor management and to antihypertensive pharmacotherapy, not a substitute for either [6,8]. The prescription that best fits current evidence is therefore supervised or telemonitored aerobic training, supplemented by resistance, isometric, or respiratory-muscle work when appropriate. Training should be sustained indefinitely and adjusted to the patient’s pressure phenotype, comorbidity, safety profile, and adherence constraints.

## 6. Gaps and Future Directions

In hypertensive heart disease, the endpoints used to judge exercise training are mismatched to the mechanisms it engages. Resting readouts (E/e′, left ventricular mass index, and pulse-wave velocity) often show no change even when functional capacity improves [23,29]. Whether that reflects an absent effect or an insensitive measurement is unresolved, and the trials as designed cannot separate the two. Provocative and tissue-level imaging offers a way to separate them. Exercise-stress echocardiography unmasks the impaired filling reserve resting Doppler misses, left atrial strain captures diastolic burden as an early load-sensitive marker, and cardiac magnetic resonance extracellular-volume mapping quantifies the diffuse fibrosis conventional indices cannot resolve [5,96,97,98]. Trials should prespecify such modalities as endpoints, so that the question of whether remodeling changes at all can be separated from the question of whether resting imaging would register it.

Whether exercise training improves hard outcomes in hypertensive heart disease remains unresolved. Updated HFpEF meta-analysis suggests that exercise-based cardiac rehabilitation may reduce rehospitalization risk. The signal is nonetheless pooled across phenotype-mixed HFpEF trials, mortality remains neutral, and no event-driven trial has tested hospitalization or survival in hypertensive heart disease specifically [92,93,100]. A second gap concerns the population studied. No HFpEF exercise trial has enrolled an etiologically restricted hypertensive cohort. The flagship trials are 84–86% hypertensive, and up to 93% in one substudy, but all are phenotype-mixed. Even the surrogate efficacy estimates are therefore not cleanly specific to hypertensive heart disease, a shortfall a dedicated position statement on exercise in hypertensive HFpEF also stresses [17]. The field needs multicentre event-driven trials stratified by hypertensive phenotype and designed around adjudicated hospitalization, cardiovascular death, and all-cause mortality.

The exercise prescription for hypertensive heart disease remains undifferentiated. Uniform aerobic, resistance, and isometric recommendations overlook that structurally, vascularly, or autonomically dominant patients may each respond best to a different modality [6,8,17]. Whether home-based, telehealth-delivered programs preserve the autonomic and vascular adaptations achieved under supervision is largely untested in hypertensive cohorts. Phenotype-guided, delivery-pragmatic trials should match modality to the dominant lesion and adopt ambulatory rather than office pressure as the primary signal.

The exercise-training evidence in hypertensive heart disease also generalizes poorly. Many smaller mechanistic and structural trials remain single-centre, modest in size, sex-skewed, short, and selective (Supplementary Materials Table S1) [42,80,92,93], and the multimorbid patients who dominate real-world practice are often excluded. Trials should recruit such populations deliberately and prespecify subgroup analysis by sex, age, race and ethnicity, and comorbidity burden.

Individual response to exercise training in hypertension is heterogeneous and deserves explicit study rather than averaging away. A substantial subset of patients show little or no fall in pressure after training, and the determinants of non-response, whether genetic, autonomic, or related to baseline RAAS activity, remain unknown [41,48,112]. Trials should report responder distributions rather than group means alone and pair them with biomarker and autonomic profiling. Identifying non-responders early would sharpen exercise from a population-level recommendation toward individualized therapy.

## 7. Conclusions

Exercise training belongs in the routine management of hypertensive heart disease, as an addition to antihypertensive pharmacotherapy and comprehensive risk-factor treatment rather than a substitute for either [6,8]. The supporting trials enrolled hypertensive or predominantly hypertensive cohorts rather than cohorts defined by established HHD. The recommendation rests on consistent effects in three domains. Clinic and ambulatory blood pressure fall, functional capacity rises, and quality of life improves in HFpEF cohorts in which hypertension predominates [9,23,29]. Autonomic and endothelial indices also respond, although as measured responses rather than as demonstrated mediators of cardiac benefit.

What exercise has not been shown to do deserves equal clarity. No human trial in hypertension has demonstrated regression of myocardial fibrosis. Favourable change in left ventricular geometry appears in small trials in mild or high-normal hypertension and tracked the achieved blood-pressure fall wherever the relationship was tested. No independent structural effect is established in HHD, and mass regression itself is inconsistent. Vascular endpoints improve unevenly across compartments, and because carotid–femoral pulse-wave velocity varies with distending pressure, the available trials do not establish intrinsic arterial destiffening. Resting diastolic indices change inconsistently even where exercise capacity improves reliably. No trial has been powered for clinical events in an etiologically defined HHD population, and the one meta-analysis reporting lower rehospitalization in HFpEF pooled phenotype-mixed cohorts and found no mortality benefit [100].

The practical consequence is a prescription that can be confident about dose and should stay modest about mechanism. Moderate aerobic training remains first-line, resistance and isometric work are legitimate additions, and the dose should be sustained, since gains from at least the isometric and inspiratory modalities regress on detraining [8,80,105]. Phenotype-guided selection is a reasonable direction rather than an evidence-based algorithm, because no trial has yet randomized patients to a modality chosen by their dominant structural, vascular, or autonomic lesion.

Closing the remaining gaps requires trials that enrol hypertensive patients by cardiac phenotype rather than by convenience. Such trials should report vascular endpoints by compartment with pressure adjustment, measure diastolic function under load, use tissue characterization to test fibrosis directly, and be powered for adjudicated clinical events. Until such trials report, exercise training in HHD is well supported as a physiological and blood-pressure intervention and remains unproven as a modifier of hard outcomes.

## Figures and Tables

**Table 1:** Clinical evidence map for exercise training across the HHD continuum. Outcomes are grouped by phenotype and endpoint rather than placed on a common effect-size scale. Abbreviations: BP, blood pressure; cfPWV, carotid–femoral pulse wave velocity; FMD, flow-mediated dilation; HFpEF, heart failure with preserved ejection fraction; HHD, hypertensive heart disease; LV, left ventricular; PWV, pulse wave velocity.

Domain	Evidence Class and Population	More Consistent Finding	Interpretive Boundary
BP and autonomic regulation	Hypertension-specific supportive: pre-hypertension, uncomplicated, and resistant hypertension trials	Clinic and ambulatory BP fall with training added to usual care [8,9]; selected studies report lower sympathetic activity or improved baroreflex indices.	No HHD-specific structural benefit is established. Office and ambulatory BP are not interchangeable.
Vascular function and load	Hypertension-specific supportive: elevated-BP to stage-1 hypertensive cohorts studied with FMD, microvascular flow, central BP, wave reflection, or regional PWV	Endothelial and microvascular endpoints improve; BP reduction is more consistent than cfPWV change [24,25,26].	Vascular beds and endpoints are non-interchangeable. PWV is pressure-dependent, so its reduction alone does not establish structural arterial remodeling.
LV geometry, mass, and fibrosis	Hypertension-specific supportive: small trials in high-normal, elevated, or mild hypertension rather than established HHD; mechanistic support is preclinical	Some trials report improved relative wall thickness or LV mass [27,28].	Established-HHD evidence is sparse, and direct human evidence for fibrosis regression is absent.
Diastolic function and HFpEF	Predominantly hypertensive mixed: mixed-etiology HFpEF trials, not HHD-restricted cohorts	Exercise capacity improves more consistently than resting cardiac indices [23,29].	Resting diastolic changes are inconsistent, and HHD-specific effects on hospitalization and mortality remain uncertain.

**Table 3:** Practical FITT starting framework and evidence boundaries. The aerobic and dynamic resistance doses follow consensus recommendations; the isometric and inspiratory doses reproduce the protocols of the cited hypertension trials; the interval dose reproduces a trial in predominantly hypertensive HFpEF. All should be individualized after clinical and BP assessment.

Modality	Practical Starting Dose	Evidence Boundary
Moderate aerobic	3–5 days/week; 150–300 min/week; about 40–59% heart-rate reserve or Borg 12–13; progress gradually	First-line recommendation with the strongest ambulatory-BP evidence; HHD-specific structural and event evidence is limited [8,9].
Dynamic resistance	2–3 days/week; 1–3 sets of 8–12 repetitions at low-to-moderate load; avoid breath-holding	Supports BP control, strength, and adherence; no established LVH-regression advantage [8,104].
Isometric	3 days/week; 4 bouts of 2 min with 1–4 min rest, about 11–20 min/session; handgrip at 30% maximum voluntary contraction, or wall squat at a knee angle giving about 95% of peak heart rate; check pre-session BP	Strong hypertension BP signal, but established-HHD, long-term safety, and event evidence remain sparse [10,12,80].
HIIT	3 days/week; 4 intervals of 4 min at 80–90% heart-rate reserve with active recovery, about 38 min/session; only after stabilization and familiarization, preferably supervised	No demonstrated capacity advantage over moderate continuous training in predominantly hypertensive HFpEF [93].
Inspiratory muscle training	5 days/week; 30 breaths/session at 75% of maximal inspiratory pressure, about 8 min/session; supplements rather than replaces aerobic and resistance work	Time-efficient, but evidence is short-term and benefit can regress after detraining [105].

## Data Availability

No new patient-level data were created or analyzed in this study. The aggregate evidence summarized in this review is available in the cited publications.

## Supplementary Materials

The following supporting information can be downloaded at: https://www.mdpi.com/article/10.3390/jcdd13090441/s1, Table S1: Structured design and reporting limitations of key human studies. The entries identify limitations relevant to interpretation and are not domain-level risk-of-bias judgments, exercise-reporting scores, or certainty grades; Table S2: Representative PubMed search strategies retained during review development and last rerun on 10 July 2026. Additional targeted searches and citation tracing were used to update individual endpoints and identify cited reports.
