# Supplementary material for: Exercise Training Across the Hypertensive Heart Disease Continuum: Clinical Evidence and Implications for Prescription
Source: J Cardiovasc Dev Dis. 2026 Sep 7;13(9):441. doi: 10.3390/jcdd13090441 (PMC13607069; doi:10.3390/jcdd13090441)
Supplement: Supplementary file 1 [file jcdd-13-00441-s001.zip › jcdd-4464023-supplementary.pdf]

# Supplementary Materials

## Exercise Training across the Hypertensive Heart Disease Continuum: Clinical Evidence and Implications for Prescription

Xiongkai Yu[1,2,3], Chi Zhang[1], Lu Liu[1] and Huimin Chen[1,2,3,*]

[1] Department of Cardiology, The Second Affiliated Hospital, School of Medicine, Zhejiang University, Hangzhou 310009, China

[2] State Key Laboratory of Transvascular Implantation Devices, Hangzhou 310009, China

[3] Heart Regeneration and Repair Key Laboratory of Zhejiang Province, Hangzhou 310009, China
Correspondence: daniel_2020@zju.edu.cn (H.C.)

# Supplementary Table S1

**Table S1:** Structured design and reporting limitations of key human studies. The entries identify limitations relevant to interpretation and are not domain-level risk-of-bias judgments, exercise-reporting scores, or certainty grades.

| Study | Design | Design or reporting limitation |
|---|---|---|
| Rinder 2004 | Controlled training trial | Approximately 50% dropout in exercise arm; no intention-to-treat analysis; small completed sample |
| Hinderliter 2002 | Randomized controlled trial | 29% non-interpretable echocardiograms; exercise and weight loss co-intervention; LVMI trend only |
| O'Driscoll 2022 | Randomized controlled trial | Small sample; White men only; four-week intervention and reversible effects |
| Rodrigues-da-Silva 2025 | Controlled telerehabilitation trial | Small sample; underpowered echocardiographic outcomes; 2:1 group imbalance; preliminary design |
| Schultz 2011 | Randomized lifestyle trial | Combined diet and exercise; hypertensive exercise response was secondary; diabetes cohort rather than defined HHD |
| Sjurdarson 2024 | Controlled training studies | Short duration; women only; echocardiographic outcomes; baseline age difference in one comparison |
| Edelmann 2011 | Randomized controlled pilot | Small pilot; supervised combined intervention; short follow-up; no clinical-event assessment |
| Edelmann 2025 | Randomized controlled trial | Open-label intervention; incomplete adherence; hierarchical primary endpoint not met |
| Mueller 2021 | Randomized controlled trial | Staff not blinded; declining home-phase adherence; primary capacity change below prespecified MCID |
| Gevaert 2025 | Randomized substudy | Small, selected substudy; moderate and interval training groups pooled; limited power for mediation |
| Sowa 2022 | Randomized mechanistic substudy | Very small groups; single site; biomarker endpoints; supervised-phase signal not sustained |
| Sugita 2023 | Non-randomized controlled trial | Open-label design; baseline imbalance; mixed-etiology HFpEF cohort |
| Edwards 2026 | Randomized controlled trial | Four-week, 38-patient study; powered for BP rather than long-term efficacy or safety |
| Lopes 2021 | Randomized controlled trial | Modest sample; aerobic training only; not powered for subgroup effects |
| Lopes 2023 | Secondary randomized-trial analysis | Primary trial powered for 24-h systolic BP; biomarker analyses underpowered |
| Banks 2024 | Controlled training trial | 26 participants; nine weeks; no preregistration; hypertension based on screening BP |
| Cahu Rodrigues 2020 | Controlled training study | Small sample; responder-only analysis; standard errors rather than standard deviations reported |
| Craighead 2021 | Randomized pilot trial | 18 participants per group; six weeks; predominantly White cohort; limited duration for arterial remodeling |
| Blumenthal 2010 | Randomized lifestyle trial | Small, selected sample; exercise, weight loss, and diet components; no clinical-event power |
| Pedralli 2020 | Randomized exercise comparison | No non-exercise control; small sample; eight weeks; mechanistic interpretation indirect |
| Toninelli 2026 | Within-subject longitudinal study | 24 participants; no randomized parallel control; six-week intervention; improvements reversed after detraining |
| Dimeo 2012 | Randomized controlled trial | Open-label; baseline age difference; self-reported compliance; no FMD measurement |
| Beck 2013 | Randomized controlled trial | Small groups; eight weeks; not powered for sex or race effects; no autonomic measures |
| Blumenthal 2021 | Randomized controlled trial | Single site; missing FMD and BRS data; four months; no clinical-event power |
| Laterza 2007 | Randomized controlled trial | Small sample; no sham or attention control; single centre |
| Brito 2024 | Randomized crossover/controlled study | Very small MSNA subgroup; no non-exercise control; sympathetic-BRS result $P = 0.052$ |
| Taylor 2018 | Randomized controlled trial | Four weeks; White men only; unsupervised; spontaneous-sequence BRS; no direct MSNA |
| Oliveira-Dantas 2020 | Controlled training study | Small sample; single centre; women only; BP pharmacologically controlled |
| Collier 2009 | Randomized exercise comparison | Small sample; four weeks; no non-exercise control; indirect autonomic indices |

LVMI, left ventricular mass index; HHD, hypertensive heart disease; HFpEF, heart failure with preserved ejection fraction; MCID, minimal clinically important difference; BP, blood pressure; FMD, flow-mediated dilation; BRS, baroreflex sensitivity; MSNA, muscle sympathetic nerve activity.

# Supplementary Table S2

**Table S2:** Representative PubMed search strategies retained during review development and last rerun on 10 July 2026. Additional targeted searches and citation tracing were used to update individual endpoints and identify cited reports.

| **Question** | **Saved PubMed search string** |
|---|---|
| Core exercise/HHD map | ("Hypertension"[Mesh] OR "Hypertrophy, Left Ventricular"[Mesh]) AND ("Cardiac Rehabilitation"[Mesh] OR "Exercise Therapy"[Mesh] OR "Exercise"[Mesh]) AND ("2020"[PDAT]:"2026"[PDAT]) |
| LV structure | ((exercise training[Title/Abstract] OR cardiac rehabilitation[Title/Abstract]) AND (hypertension[Title/Abstract] OR hypertensive[Title/Abstract]) AND (left ventricular mass[Title/Abstract] OR LVMI[Title/Abstract] OR ventricular remodeling[Title/Abstract] OR hypertrophy[Title/Abstract]) AND (randomized[Title/Abstract] OR trial[Title/Abstract] OR controlled[Title/Abstract])) |
| Diastolic function/HFpEF | ((exercise training[Title/Abstract] OR cardiac rehabilitation[Title/Abstract]) AND (diastolic function[Title/Abstract] OR E/e[Title/Abstract] OR filling pressure[Title/Abstract] OR diastolic dysfunction[Title/Abstract]) AND (hypertension[Title/Abstract] OR hypertensive[Title/Abstract] OR HFpEF[Title/Abstract] OR preserved ejection fraction[Title/Abstract]) AND (randomized[Title/Abstract] OR trial[Title/Abstract] OR meta-analysis[Publication Type])) |
| Fibrosis/tissue characterization | ((exercise training[Title/Abstract] OR cardiac rehabilitation[Title/Abstract]) AND (hypertension[Title/Abstract] OR hypertensive[Title/Abstract] OR HFpEF[Title/Abstract]) AND (fibrosis[Title/Abstract] OR extracellular volume[Title/Abstract] OR ECV[Title/Abstract] OR T1 mapping[Title/Abstract] OR cardiac magnetic resonance[Title/Abstract])) |
| Clinical events | ((exercise training[Title/Abstract] OR cardiac rehabilitation[Title/Abstract]) AND (HFpEF[Title/Abstract] OR preserved ejection fraction[Title/Abstract] OR hypertensive heart disease[Title/Abstract]) AND (hospitalization[Title/Abstract] OR mortality[Title/Abstract] OR death[Title/Abstract] OR rehospitalization[Title/Abstract]) AND (randomized[Title/Abstract] OR trial[Title/Abstract] OR meta-analysis[Publication Type])) |
| Autonomic outcomes | (hypertension[mh] OR hypertensive[Title/Abstract]) AND (heart rate variability[Title/Abstract] OR muscle sympathetic nerve activity[Title/Abstract] OR baroreflex sensitivity[Title/Abstract] OR autonomic[Title/Abstract]) AND (exercise training[Title/Abstract] OR cardiac rehabilitation[Title/Abstract] OR aerobic exercise[Title/Abstract]) AND (randomized controlled trial[Publication Type] OR clinical trial[Publication Type]) NOT review[Publication Type] |
| Arterial stiffness | exercise training AND ("pulse wave velocity" OR "arterial stiffness") AND hypertens* AND randomized |
